# Large variations in atrial fibrillation screening practice after ischemic stroke and transient ischemic attack in Sweden: a survey study

**DOI:** 10.1186/s12883-024-03622-2

**Published:** 2024-04-11

**Authors:** Kajsa Strååt, Eva Isaksson, Ann Charlotte Laska, Elisabeth Rooth, Emma Svennberg, Signild Åsberg, Per Wester, Johan Engdahl, on behalf of AF SPICE steering committee

**Affiliations:** 1 Department of Clinical Sciences, Karolinska Institutet, Danderyd Hospital, Stockholm, Sweden; 2 Department of Cardiology, Danderyd Hospital, Stockholm, SE-182 88 Sweden; 3 Department of Medicine, Karolinska Institutet, Karolinska University Hospital Huddinge, Stockholm, Sweden; 4 Department of Medical Sciences, Uppsala University, Uppsala, Sweden; 5 Department of Public Health and Clinical Medicine, Umeå University, Umeå, Sweden

**Keywords:** Atrial fibrillation, Screening, Ischemic stroke, Transient ischemic attack, Inpatient telemetry ECG, Holter ECG, Handheld ECG, Event loop recorder, Implantable loop recorder

## Abstract

**Background:**

Atrial fibrillation (AF) screening after ischemic stroke or transient ischemic attack (TIA) is given high priority in clinical guidelines. However, patient selection, electrocardiogram (ECG) modality and screening duration remains undecided and current recommendations vary.

**Methods:**

The aim of this study was to investigate the clinical practice of AF screening after ischemic stroke or TIA at Swedish stroke units. In collaboration with the stakeholders of *the Swedish Stroke Register* (Riksstroke) a digital survey was drafted, then tested and revised by three stroke consultants. The survey consisted of 17 multiple choice/ free text questions and was sent by e-mail to the medical directors at all stroke units in Sweden.

**Results:**

All 72 stroke units in Sweden responded to the survey. Most stroke units reported that ≥ 75% of ischemic stroke (69/72 stroke units) or TIA patients (67/72 stroke units), without previously known AF, were screened for AF. Inpatient telemetry ECG was the method of first-choice in 81% of the units, but 7% reported lack of access. A variety of standard monitoring durations were used for inpatient telemetry ECG. The second most common choice was Holter ECG (17%), also with considerable variations in monitoring duration. Other AF screening modalities were used as a first-choice method (handheld and patch ECG) but less frequently.

**Conclusions:**

Clinical practice for AF screening after ischemic stroke or TIA differed between Swedish stroke units, both in choice of AF screening methods as well as in monitoring durations. There is an urgent need for evidence and evidence-based recommendations in this field.

**Trial registration:**

Not applicable.

**Supplementary Information:**

The online version contains supplementary material available at 10.1186/s12883-024-03622-2.

## Background

Ischemic stroke is one of the leading causes of mortality worldwide and a major cause of permanent disability in adults [1]. The management of stroke patients has improved significantly over the last decades and designated stroke units have played an important role [2]. In Sweden, over the last 20 years, a majority of stroke patients are cared for at a dedicated stroke unit [3]. Since 1994, *the Swedish Stroke Register* (Riksstroke) has collected data on morbidity and care of stroke patients, with the addition of transient ischemic attack (TIA) patients in 2010 [4]. Most individuals suffering from cerebrovascular events in Sweden are admitted to hospital, where 92% of stroke patients and 81% of TIA patients are treated at a stroke unit [5]. All hospitals admitting patients with acute stroke and TIA in Sweden are reporting to the register [4].

Atrial fibrillation (AF), the most prevalent clinically relevant arrhythmia, is associated with increased risk of ischemic stroke [6, 7] and with increased mortality among stroke patients [8]. Oral anticoagulant (OAC) treatment markedly reduces this risk [9]. Since AF is often paroxysmal and asymptomatic [10], initial AF screening with at least 24 h electrocardiogram (ECG) monitoring (recommended by most societies) after ischemic stroke and TIA is given high priority in national and international guidelines [11–16]. However, patient selection, ECG modality, and screening duration remain undecided, and clinical guidelines vary [11–17]. By the time this study was conducted, the Swedish national board of health and welfare recommended that AF screening for 24–48 h, with Holter ECG or inpatient telemetry ECG, should be part of the routine care at Swedish stroke units [18]. The recommendations also stated that monitoring durations over 48 h should be considered for selected patients with suspected embolic stroke. Use of handheld ECG monitoring and implantable loop recorders (ILR) was only recommended in research projects. The guidelines for cardiac monitoring in ischemic stroke and TIA patients from European Society of Cardiology, European Stroke Organisation, American heart association/American stroke association, at the time of this study, are summarized in Table 1.

**Table 1:** Summary of relevant guidelines for AF screening after ischemic stroke or TIA, in force at the time of the survey (November 2021)

Recommendation	COR	LOE
**2020 ESC Guidelines for the diagnosis and management of atrial fibrillation** [13]		
In patients with acute ischaemic stroke or TIA and without previously known AF, […] short-term ECG recording for at least the first 24 h, followed by continuous ECG monitoring for at least 72 h whenever possible [is recommended]	1	B
In selected stroke patients without previously known AF, additional ECG monitoring using long-term non-invasive ECG monitors or insertable cardiac monitors should be considered, to detect AF	2a	B
**AHA/ASA Guideline 2021 Guideline for the Prevention of Stroke in Patients With Stroke and Transient Ischemic Attack** [14]		
In patients suspected of having a stroke or TIA, an ECG is recommended to screen for AF and atrial flutter and to assess for other concomitant cardiac conditions	1	B-R
In patients with cryptogenic stroke who do not have a contraindication to anticoagulation, long-term rhythm monitoring with mobile cardiac outpatient telemetry, ILR, or other approach is reasonable to detect intermittent AF	2a	B-R
**Guidelines for the Early Management of Patients With Acute Ischemic Stroke: 2019 Update to the 2018 Guidelines for the Early Management of Acute Ischemic Stroke: A Guideline for Healthcare Professionals** [19]		
Cardiac monitoring is recommended to screen for AF and other potentially serious arrhythmias that would necessitate emergency cardiac interventions. Cardiac monitoring should be performed for at least the first 24 h	1	B-NR
The effectiveness of prolonged cardiac monitoring during hospitalization after acute ischemic stroke to guide treatment selection for prevention of recurrent stroke is uncertain	2b	C-LD
**Guideline: Consensus statements and recommendations from the ESO-Karolinska Stroke Update Conference, Stockholm 11–13 November 2018**[20] What is good clinical practice in work up for suspected cardio-embolic cases?		
[…] 24-h 12-lead ECG		A
Continuous monitoring of heart rhythm up to 30 days is reasonable in patients with embolic stroke of undetermined aetiology despite recommended diagnostic work up to increase covert AF detection		A
It remains to be firmly established that the increased detection of brief episodes of AF will lead to a reduction in stroke recurrence after adequate treatment		C

Table 1 Detailed version, with definitions, is available in Additional file 1

Several randomised controlled trials (RCT(s)) have shown that the AF yield of extended ECG monitoring after ischemic cerebrovascular events is higher than that of standard of care [21–24]. One RCT, powered to detect differences in OAC treatment 12 months after index stroke, demonstrated neutral results [24]. No study has investigated the potential benefits of AF screening after ischemic stroke or TIA in terms of reduced stroke recurrence and mortality as their primary endpoint. A meta-analysis [25] that pooled data from 5 RCTs and 3 observational studies, covering extended ECG monitoring in ischemic stroke and TIA patients, showed lower recurrence of stroke during follow-up in the analysis for the observational studies but not for the RCTs. To this day, there is no solid evidence for clinical benefits of prolonged ECG monitoring after ischemic stroke and TIA. How clinicians should treat AF detected with prolonged screening after stroke or TIA is a subject for ongoing and future research [26].

In Sweden, *the Swedish Stroke Register* [5] reports a high average proportion of screening for AF, with at least 24 h ECG monitoring, in AF naïve patients with ischemic stroke or TIA during the acute hospital stay. However, there are discrepancies in the proportion of patients that are screened at the different stroke units and there are no data on methods used for AF screening, nor on monitoring durations.

## Methods

### Aim

The aim of this study was to investigate the clinical practice of AF screening after ischemic stroke or TIA at Swedish stroke units.

### Development of the survey

In collaboration with the stakeholders of *the Swedish Stroke Register* the most important variables to study were identified: AF screening coverage and ECG modality, first choice of ECG modality, ECG monitoring duration, repeated AF screening routines and clinical follow-up.

A digital survey was drafted in REDCap (version 11.1.15, Vanderbilt University, Nashville, TN, USA), then evaluated and revised by three stroke consultants (AC.L., S.Å., E.R.) of which two also contributed with comments using “Think aloud” i.e. observation while verbalising their thoughts when answering the survey [27]. The survey consisted of 16 multiple choice questions with the possibility of free text comments and one free text question (Additional file 2). In the first two questions the medical directors of the stroke unit were asked to estimate the proportion of patients, without known AF, being screened for AF at their stroke unit choosing from 4 alternatives: <50%, 50–74%, 75–94% or ≥95%. As opposed to data collected yearly from *the Swedish Stroke Register* on ECG monitoring ≥ 24 h, we did not define a lower monitoring duration limit. In these questions we separated ischemic stroke patients and TIA patients. In all the other questions we did not ask for data on ischemic stroke and TIA patients separately. The last five questions in the survey were not related to AF screening routines and thus left out in the statistical analysis (results are presented in Additional file 3).

### Distribution of the survey

The contact information to all 72 stroke units in Sweden was provided by *the Swedish Stroke Register* office. The survey was sent by e-mail to the medical directors of all stroke units in November 2021.

### Statistical analysis

The results are presented with descriptive statistics. For the comparison between hospital types and patient volume groups we used chi-square or Fisher exact test as appropriate. P-values < 0.05 were considered significant. All analyses were performed in Stata/SE 17.0 (Stata corp, College Station, TX, USA).

For stratified data analyses by hospital type the 72 stroke units were divided into university hospitals, large non-university hospitals and small non-university hospitals [28]. To avoid small numbers, university hospitals and large non-university hospitals were merged to one group. Two equal groups according to patient volume (small and large) at the stroke units were also compared. Patient volume was determined by number of registrations per stroke unit in *the Swedish Stroke Register* in 2021 [5], i.e. registered hospitalisations for stroke or TIA during that year. In multiple choice questions with three or four alternatives, data were merged creating two alternatives to get a more apprehensible comparison between the groups (e.g. for questions about proportion of screened patients < 50% and 50–74% were merged to < 75%, 75–94% and ≥ 95% were merged to ≥ 75%).

## Results

The digital survey was submitted to the medical directors of all Swedish stroke units on November 16th 2021 with regular e-mail reminders until March 7th 2022. The response rate was 100% (*n* = 72) to the survey and all questions except one (regarding other AF screening methods used) were answered by all participants if applicable. In median, the time to respond to the survey was 16 days and all replies were collected by an individual URL-link connected to REDCap.

### Proportion of AF screened patients

Of all stroke units, 58% (42/72) reported screening coverage of ≥ 95% of AF naïve ischemic stroke patients while 96% (69/72) reported that at least 75% were screened. In TIA patients, 61% (44/72) reported screening coverage of ≥ 95%, whereas 93% (67/72) reported that at least 75% were screened for AF. AF screening in less than 50% of ischemic stroke patients was stated from one stroke unit, for TIA patients this was stated from two stroke units.

### AF screening methods and availability

**Fig. 1:**
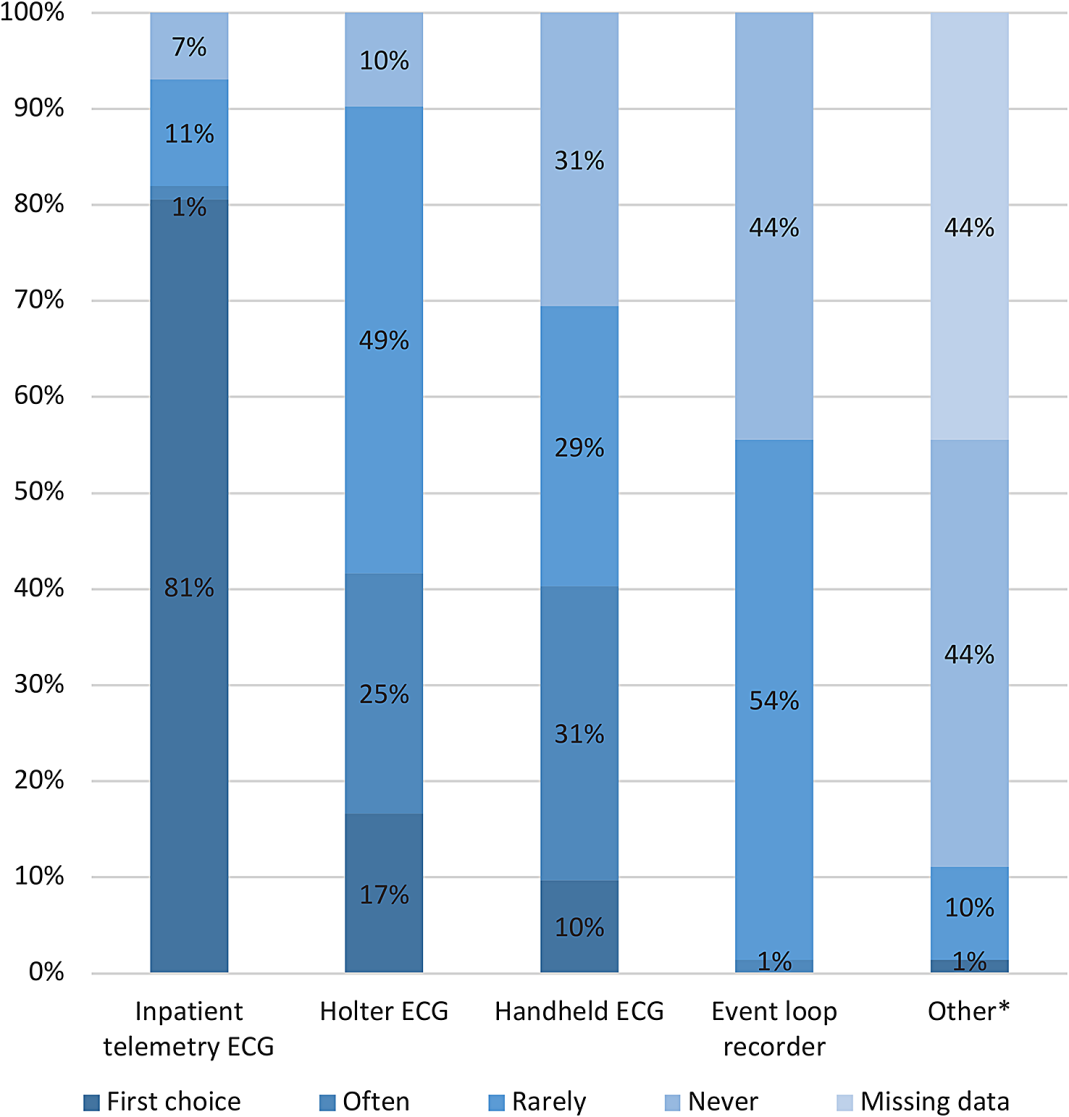
Use of different AF screening methods at Swedish stroke units (*n* = 72). *Other methods used were patch ECG (first choice at one stroke unit) and ILR

**Fig. 2:**
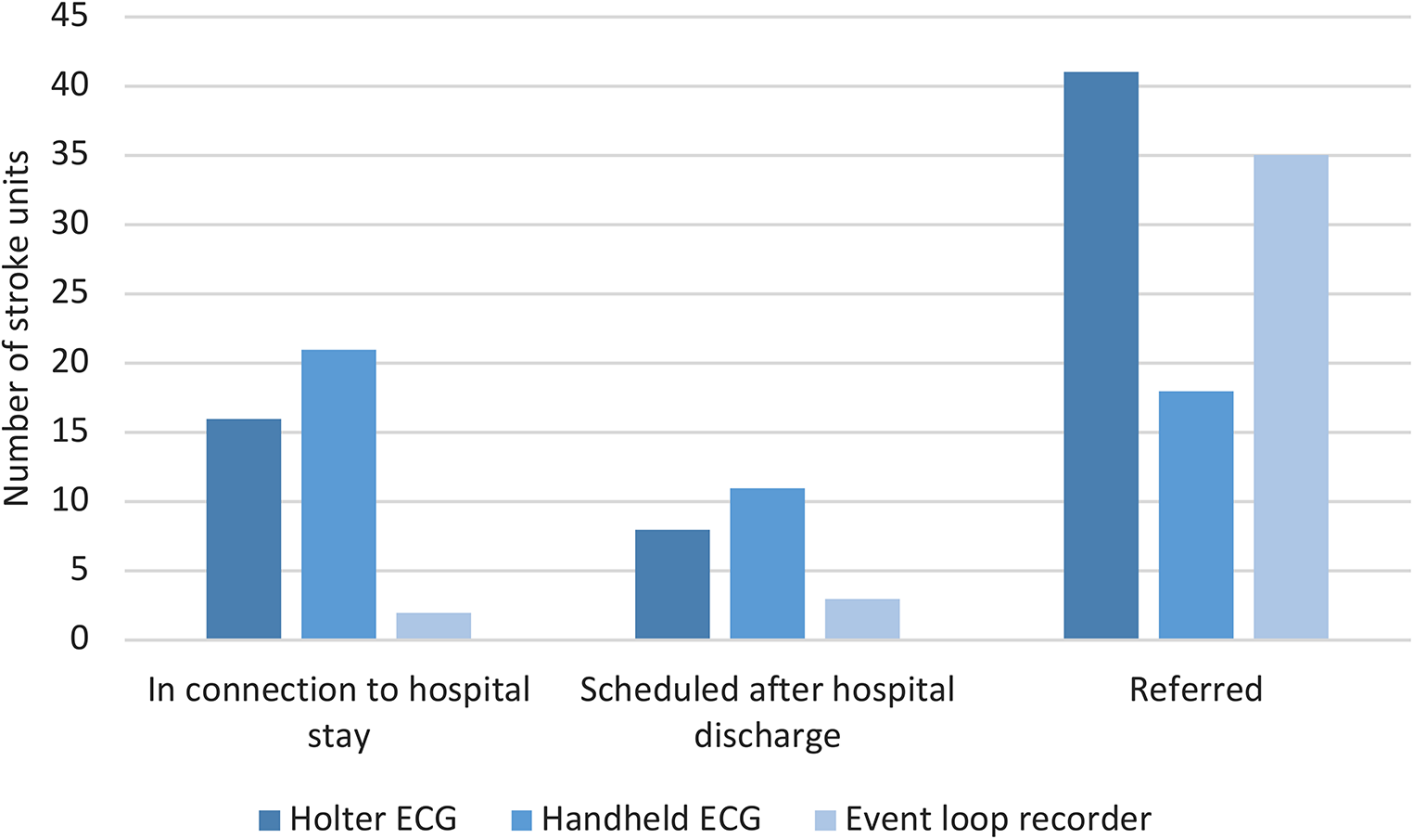
Time for start of ECG recording according to the stroke unit’s practice for Holter ECG, handheld ECG and event loop recorder

Inpatient telemetry ECG was the/one of the first-choice method(s) for AF screening at 81% (58/72) of the stroke units, while 7% (5/72) reported no availability of this method (Fig. 1). Holter ECG and handheld ECG were also frequently used for AF screening. ECG modalities with longer continuous monitoring duration such as event loop recorder (ELR), patch ECG and ILR were rarely used. For the most frequently used non-telemetry modalities some of the stroke units stated start of ECG recordings in connection to hospital stay (Fig. 2). This routine was most common when using handheld ECG, which was applied by 21/72 stroke units. However, for Holter ECG and ELR a majority of the stroke units referred their patients for investigation after hospital discharge. A minority of the stroke units scheduled an appointment, that was not in direct connection to hospital stay, for start of the ECG recording.

### ECG monitoring durations

**Fig. 3:**
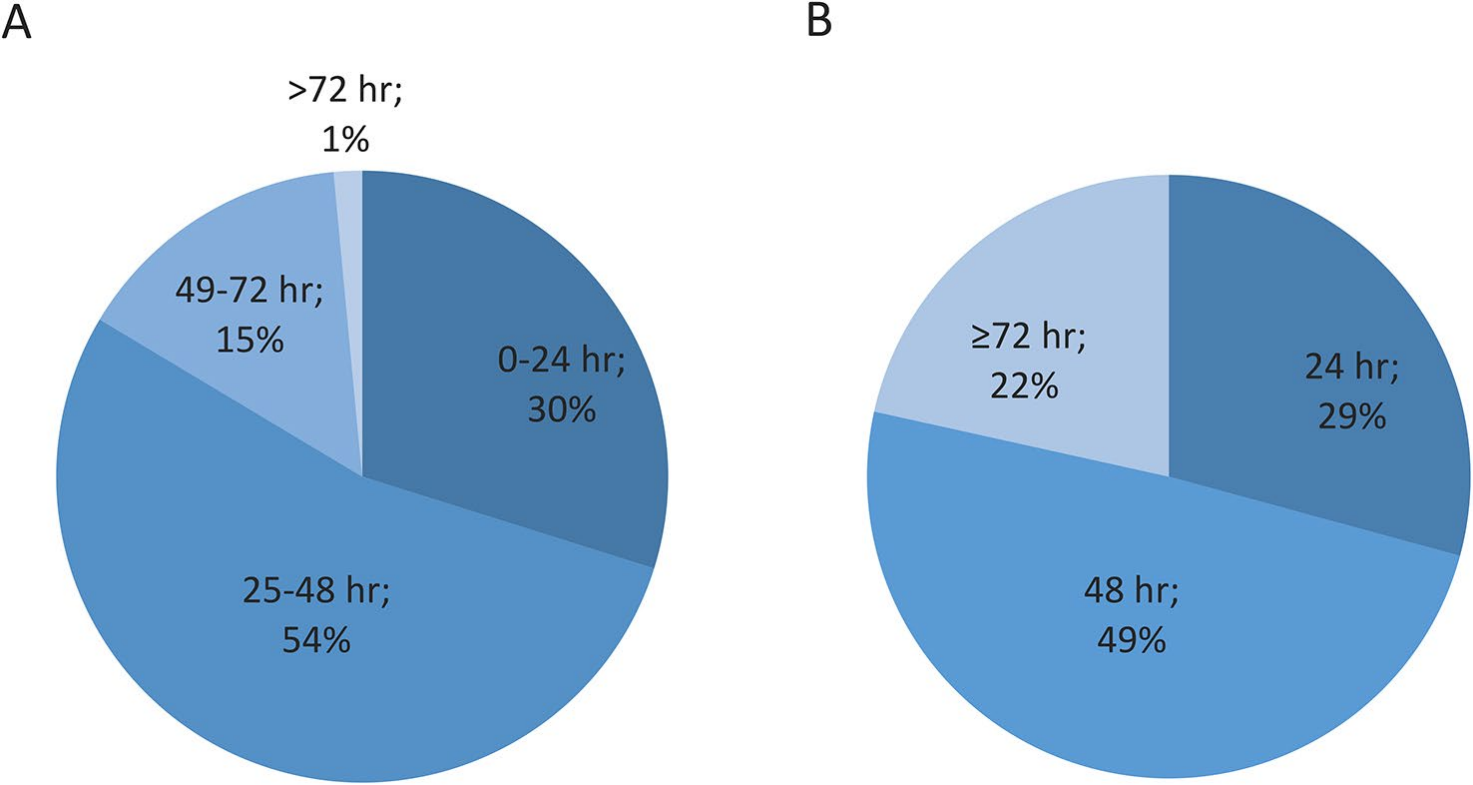
(**A**) Inpatient telemetry ECG standard monitoring durations (**B**) Holter ECG standard monitoring durations

ECG screening duration of 24 h or less was reported by 30% (20/67) of stroke units using inpatient telemetry ECG and by 29% (19/65) of the stroke units using Holter ECG. Of all stroke units, 13% (9/72) reported a standard monitoring duration of 24 h or less for either inpatient telemetry ECG (if not using Holter ECG) or Holter ECG (if not using inpatient telemetry ECG) or both. Approximately half of the stroke units reported that their standard monitoring durations for inpatient telemetry ECG and Holter ECG were 25–48 h and 48 h respectively (Fig. 3A and B).

Repeated ECG monitoring in case of high suspicion of cardiac embolisation was reported by 86% of the stroke units, preferably with repeated Holter ECG (73%) or handheld ECG (48%). ELR (16%), ILR (6%) and patch ECG (2%) were more infrequently used for this purpose.

### Inpatient telemetry ECG reading

Reading of inpatient telemetry ECG was routinely performed by the responsible physician for the patient at the stroke unit (78% of the reporting stroke units), by a cardiologist (7%), by the nurse responsible for the patient at the stroke unit (4%) or by others (10%). In stroke units with non-cardiologist ECG reading, 21% (13/62) of the stroke units reported use of cardiologist consultation on regular basis.

### Results stratified by hospital type and patient volume

Out of all 72 stroke units, 31 were university hospitals (*n* = 9) plus large non-university hospitals (*n* = 22) and 41 were small non-university hospitals. There were no significant differences in clinical practice, addressed by this survey, between the hospital type groups (Additional file 4).

Division of the stroke units into patient volume groups resulted in two equal groups with 36 stroke units in each. There was a significant difference in the use of handheld ECG where large patient volume stroke units used this method more frequently than small patient volume stroke units (53% vs. 25% reported “often” or “first choice”, p 0.016). There were no other significant differences in clinical practice between the patient volume groups (Additional file 4).

## Discussion

This survey of all 72 Swedish stroke units showed large variations in clinical practice for AF screening after ischemic stroke and TIA. Inpatient telemetry ECG was the most common first-choice of screening method, with Holter ECG and handheld ECG, the second and third most common first-choice. Overall, there was a large span of different monitoring durations and methods used. These differences could not be explained by hospital type, nor by different patient volumes, except for a more frequent use of handheld ECG at stroke units with large patient volumes. When it comes to monitoring duration, 13% reported a standard monitoring duration of 24 h or less for either inpatient telemetry ECG (if not using Holter ECG) or Holter ECG (if not using inpatient telemetry ECG) or both, which is in the lower interval or below nationally recommended screening durations at the time of the study.

The relevance of timing of out of hospital ECG monitoring remains unclear and the clinical significance of AF detection in different post-stroke periods is not established [26]. A meta-analysis [29], investigating the yield of AF by ILR monitoring in ischemic stroke patients, could not detect an association between AF yield and the timing of the monitoring (i.e. time from index event until ILR implantation). We observed that a majority of the stroke units referred their patients for out of hospital ECG monitoring with Holter ECG and ELR. This may delay diagnosis of AF, but the clinical significance of this delay is not established.

The variation in clinical practice may be due to the lack of evidence and consistent guidelines in this field in combination with the organisation of the Swedish healthcare system. Swedish healthcare is governed by 20 county councils, responsible for the health care of the 21 counties. Consequently, the recommendations, practices, and financial compensation models vary. The stroke units organise the AF screening in accordance with their local conditions, including availability and reimbursement for different AF screening modalities.

The effect of healthcare organisation, economic resources and local traditions are visible in an international survey [30] of diagnostic routines in cryptogenic stroke patients. The survey, with data collection in 2014, was responded to by 301 physicians involved in stroke care from 48 countries. The response rate was 30% and 82% of the responders were from high-income countries, mostly from Europe and central Asia. Diagnostic evaluations were considered routine if performed in > 75% of patients at a site. For cardiac monitoring, 100% reported routine 12-lead ECG. Inpatient telemetry ECG and 24 h Holter ECG were reported routine in 49% and 41% of the centres respectively. For cardiac monitoring longer than 24 h (Holter ECG or Loop recording) 17% reported routine use, where 0 of the 12 responding centres from low/low-middle income countries reported routine use. This study thus highlights the variations in ECG investigation standards in an international context.

A survey similar to ours was performed in Germany in 2015 [31]. The response rate of this study was lower than of ours (71.6% vs. 100%) and data not completely comparable with our study. However, the conclusions were similar, emphasising the lack of a nationwide standard for AF screening after ischemic stroke and TIA. In contrast to clinical practice at Swedish stroke units, the researchers of the German study stated that all patients admitted to German stroke units, without known AF, undergo continuous ECG monitoring to some extent. The duration of monitoring was also longer in German stroke units, where 66.8% of ischemic stroke patients and 22.2% of TIA patients were monitored for more than 48 h, whereas the corresponding proportion in our survey was 16%. A majority of the German stroke units reported the use of ILR, while only a small percentage of Swedish stroke units did. On the other hand, external event recorders were used by a higher proportion of Swedish than German stroke units (56% vs. 16.1%). Diagnostic routines in different countries may vary due to differences in reimbursement models, local tradition, and length of hospital stays. Although the data in the studies are not completely comparable, they may indicate a higher ambition regarding AF screening after ischemic stroke or TIA in Germany 2015 than in Sweden 2021. This could be an expression of Swedish stroke units being more prone to prioritize which patients to screen, possibly due to local traditions or resource limitations.

An observational cohort study [32] described the use of ECG-monitoring after ischemic stroke in Denmark. They reported that younger patients with less comorbidity and milder strokes were more often screened for AF compared to older patients with more severe strokes although the older patients were more likely to be diagnosed with AF if screened. Since AF screening is costly both in terms of clinical and economic resources it is important to optimize the patient selection. Routines for patient selection was not covered by our survey, selection mechanisms being a field for future studies.

The strengths of this study are the complete response rate, from all stroke units in Sweden, and the detailed information given by the questionnaire. However, the results are based on estimations by the medical directors at each stroke unit and does not reflect individual patient data, which is one of the study’s limitations. One finding in our study was that the inpatient telemetry ECG reading was performed by the attending physician (often stroke neurologist or stroke internist) in a majority of the stroke units. Routines for, as well as the experience of, inpatient telemetry ECG reading may differ between stroke units and attending physicians. This could affect the quality of the diagnostics from inpatient telemetry ECG. These aspects would have been interesting to study but neither ECG reading qualifications of readers nor inpatient telemetry reading routines were covered by our survey. A vast majority of all stroke and TIA patients are treated at a stroke unit [5]. Patients treated outside a stroke unit are not covered in our survey, which could lead to selection bias if the care of stroke patients outside stroke units is less adherent to stroke guidelines. There is a risk for type 2 errors, considering our relatively small sample size.

## Conclusion

Clinical practice for AF screening after ischemic stroke or TIA at Swedish stroke units showed large variations with a range of different AF screening methods and monitoring durations. A small proportion of stroke units reported ECG investigation practice in the lower interval or below national minimum recommendations. However, the large variations seen reflects the discrepancies between national and international recommendations at the time of the survey. There is an urgent need for evidence and evidence-based recommendations in this field, the present situation also implies inequality in care.

### Electronic supplementary material

Below is the link to the electronic supplementary material.

Supplementary Material 1

Supplementary Material 2

Supplementary Material 3

Supplementary Material 4

Supplementary Material 5

## Data Availability

The dataset (pseudonymised) supporting the conclusions of this article is included within the article and its additional files. Free-text answers or comments in the survey (translated to English) are available from the corresponding author upon reasonable request.

## Abbreviations

AF: Atrial fibrillation
ECG: Electrocardiogram
ELR: Event loop recorder
ILR: Implantable loop recorder
OAC: Oral anticoagulant
RCT: Randomized controlled trial
TIA: Transient ischemic attack

## References

[1] Feigin VL, Norrving B, Mensah GA (2017). Global Burden of Stroke. Circ Res.

[2] Langhorne P, Ramachandra S, Stroke Unit Trialists C (2020). Organised inpatient (stroke unit) care for stroke: network meta-analysis. Cochrane Database Syst Rev.

[3] Rudd AG, Matchar DB (2004). Health policy and outcome research in stroke. Stroke.

[4] Riksstroke. General information, https://www.riksstroke.org/general-information/ (accessed 2023-04-28).

[5] Riksstroke. Quality of the Swedish stroke care 2021, a brief summary of data for the full year 2021., https://www.riksstroke.org/wp-content/uploads/2022/11/Arsrapport-2021-engelsk-sammanfattning_final.pdf (accessed 2023-04-28).

[6] Wolf PA, Abbott RD, Kannel WB (1991). Atrial fibrillation as an independent risk factor for stroke: the Framingham Study. Stroke.

[7] Bjorck S, Palaszewski B, Friberg L (2013). Atrial fibrillation, stroke risk, and warfarin therapy revisited: a population-based study. Stroke.

[8] Marini C, De Santis F, Sacco S (2005). Contribution of atrial fibrillation to incidence and outcome of ischemic stroke: results from a population-based study. Stroke.

[9] Hart RG, Pearce LA, Aguilar MI (2007). Meta-analysis: antithrombotic therapy to prevent stroke in patients who have nonvalvular atrial fibrillation. Ann Intern Med.

[10] Flaker GC, Belew K, Beckman K (2005). Asymptomatic atrial fibrillation: demographic features and prognostic information from the Atrial Fibrillation follow-up investigation of Rhythm Management (AFFIRM) study. Am Heart J.

[11] Royal College of Physicians. National clinical guideline for stroke, Full 2016 guideline, https://www.strokeaudit.org/Guideline/Full-Guideline.aspx (accessed 2023-05-03).

[12] Group NCAFGW, Brieger D, Amerena J (2018). National Heart Foundation of Australia and the Cardiac Society of Australia and New Zealand: Australian clinical guidelines for the diagnosis and management of Atrial Fibrillation 2018. Heart Lung Circ.

[13] Hindricks G, Potpara T, Dagres N (2021). 2020 ESC guidelines for the diagnosis and management of atrial fibrillation developed in collaboration with the European Association for Cardio-Thoracic Surgery (EACTS): the Task Force for the diagnosis and management of atrial fibrillation of the European Society of Cardiology (ESC) developed with the special contribution of the European Heart Rhythm Association (EHRA) of the ESC. Eur Heart J.

[14] Kleindorfer DO, Towfighi A, Chaturvedi S et al. 2021 Guideline for the Prevention of Stroke in Patients With Stroke and Transient Ischemic Attack: A Guideline From the American Heart Association/American Stroke Association. *Stroke* 2021; 52: e364-e467. 20210524. 10.1161/STR.0000000000000375. 10.1161/STR.0000000000000375 34024117

[15] Gladstone DJ, Lindsay MP, Douketis J (2022). Canadian stroke best practice recommendations: secondary Prevention of Stroke Update 2020. Can J Neurol Sci.

[16] Rubiera M, Aires A, Antonenko K et al. European Stroke Organisation (ESO) guideline on screening for subclinical atrial fibrillation after stroke or transient ischaemic attack of undetermined origin. *Eur Stroke J* 2022; 7: VI. 20220603. 10.1177/23969873221099478. 10.1177/23969873221099478 PMC9446336 36082257

[17] Haeusler KG, Groschel K, Kohrmann M (2018). Expert opinion paper on atrial fibrillation detection after ischemic stroke. Clin Res Cardiol.

[18] Socialstyrelsen. Nationella riktlinjer för vård vid stroke, https://www.socialstyrelsen.se/globalassets/sharepoint-dokument/artikelkatalog/nationella-riktlinjer/2020-1-6545.pdf (accessed 2022-06-22).

[19] Powers WJ, Rabinstein AA, Ackerson T et al. Guidelines for the Early Management of Patients With Acute Ischemic Stroke: 2019 Update to the 2018 Guidelines for the Early Management of Acute Ischemic Stroke: A Guideline for Healthcare Professionals From the American Heart Association/American Stroke Association. *Stroke* 2019; 50: e344-e418. 20191030. 10.1161/STR.0000000000000211. 10.1161/STR.0000000000000211 31662037

[20] Ahmed N, Audebert H, Turc G et al. Consensus statements and recommendations from the ESO-Karolinska Stroke Update Conference, Stockholm 11–13 November 2018. *Eur Stroke J* 2019; 4: 307–317. 20190902. 10.1177/2396987319863606. 10.1177/2396987319863606 PMC6921948 31903429

[21] Sanna T, Diener HC, Passman RS (2014). Cryptogenic stroke and underlying atrial fibrillation. N Engl J Med.

[22] Gladstone DJ, Spring M, Dorian P (2014). Atrial fibrillation in patients with cryptogenic stroke. N Engl J Med.

[23] Wachter R, Groschel K, Gelbrich G (2017). Holter-electrocardiogram-monitoring in patients with acute ischaemic stroke (Find-AFRANDOMISED): an open-label randomised controlled trial. Lancet Neurol.

[24] Haeusler KG, Kirchhof P, Kunze C (2021). Systematic monitoring for detection of atrial fibrillation in patients with acute ischaemic stroke (MonDAFIS): a randomised, open-label, multicentre study. Lancet Neurol.

[25] Tsivgoulis G, Triantafyllou S, Palaiodimou L et al. Prolonged Cardiac Monitoring and Stroke Recurrence: A Meta-analysis. *Neurology* 2022; 98: e1942-e1952. 20220309. 10.1212/WNL.0000000000200227. 10.1212/WNL.0000000000200227 35264426

[26] Sposato LA, Field TS, Schnabel RB (2024). Towards a new classification of atrial fibrillation detected after a stroke or a transient ischaemic attack. Lancet Neurol.

[27] Fonteyn ME, Kuipers B, Grobe SJ (1993). A description of think aloud Method and Protocol Analysis. Qual Health Res.

[28] Asplund K, Sukhova M, Wester P (2015). Diagnostic procedures, treatments, and outcomes in stroke patients admitted to different types of hospitals. Stroke.

[29] Tsivgoulis G, Katsanos AH, Kohrmann M (2019). Duration of Implantable Cardiac Monitoring and Detection of Atrial Fibrillation in ischemic stroke patients: a systematic review and Meta-analysis. J Stroke.

[30] Giruparajah M, Bosch J, Vanassche T (2015). Global survey of the diagnostic evaluation and management of cryptogenic ischemic stroke. Int J Stroke.

[31] Rizos T, Quilitzsch A, Busse O (2015). Diagnostic work-up for detection of paroxysmal atrial fibrillation after acute ischemic stroke: cross-sectional survey on German stroke units. Stroke.

[32] Lyckhage LF, Hansen ML, Butt JH et al. Time trends and patient selection in the use of continuous electrocardiography for detecting atrial fibrillation after stroke: a nationwide cohort study. *Eur J Neurol* 2020; 27: 2191–2201. 20200726. 10.1111/ene.14418. 10.1111/ene.14418 32593218

