# Supplementary material for: Large variations in atrial fibrillation screening practice after ischemic stroke and transient ischemic attack in Sweden: a survey study
Source: BMC Neurol. 2024 Apr 11;24:120. doi: 10.1186/s12883-024-03622-2 (PMC11007877; doi:10.1186/s12883-024-03622-2)
Supplement: Supplementary file 1 — Supplementary Material 1 [file 12883_2024_3622_MOESM1_ESM.docx]

# Additional file 1

# Summary of relevant guidelines for AF screening after ischemic stroke or TIA, in force at the time of the survey (November 2021), with definitions

## 2020 ESC Guidelines for the diagnosis and management of atrial fibrillation^1^

In patients with acute ischaemic stroke or TIA and without previously known AF, monitoring for AF is recommended using a short-term ECG recording for at least the first 24 h, followed by continuous ECG monitoring for at least 72 h whenever possible. (Class I, level B)

In selected^c^ stroke patients without previously known AF, additional ECG monitoring using long-term non-invasive ECG monitors or insertable cardiac monitors should be considered, to detect AF. (Class IIa, level B)

^c^ Not all stroke patients would benefit from prolonged ECG monitoring; those deemed at risk of developing AF (e.g. elderly, with cardiovascular risk factors or comorbidities, indices of LA remodelling, high C2HEST score, etc.) or those with cryptogenic stroke and stroke characteristics suggestive of an embolic stroke should be scheduled for prolonged ECG monitoring

### Class of recommendation

Class I

Definition: Evidence and/or general agreement that a given treatment or procedure is beneficial, useful, effective.

Wording to use: Is recommended or is indicated

Class IIa

Definition: Weight of evidence/opinion is in favour of usefulness/efficacy

Wording to use: Should be considered

### Level of evidence

Level of evidence B: Data derived from a single randomized clinical trial or large non-randomized studies.

## AHA/ASA Guideline 2021 Guideline for the Prevention of Stroke in Patients With Stroke and Transient Ischemic Attack^2^

In patients suspected of having a stroke or TIA, an ECG is recommended to screen for atrial fibrillation (AF) and atrial flutter and to assess for other concomitant cardiac conditions. (COR 1, LOE B-R)

In patients with cryptogenic stroke who do not have a contraindication to anticoagulation, long-term rhythm monitoring with mobile cardiac outpatient telemetry, implantable loop recorder, or other approach is reasonable to detect intermittent AF (COR 2a, LOE B-R)

### Class of recommendation (COR)

Class 1 (strong)

Suggested phrases for writing recommendations:

-Is recommended

-Is indicated/useful/effective/beneficial

-Should be performed/administered/other

-Comparative-Effectiveness phrases – Treatment/strategy A is recommended/indicated in preference to treatment B – Treatment A should be chosen over treatment B

Class 2a (moderate)

Suggested phrases for writing recommendations:

-Is reasonable

-Can be useful/effective/beneficial

-Comparative-Effectiveness phrases – Treatment/strategy A is probably recommended/indicated in preference to treatment B – It is reasonable to choose treatment A over treatment B

### Level of evidence (LOE)

Level B-R (Randomized)

-Moderate-quality evidence from 1 or more RCTs

-Meta-analyses of moderate-quality RCTs

## Guidelines for the Early Management of Patients With Acute Ischemic Stroke: 2019 Update^3^

Cardiac monitoring is recommended to screen for atrial fibrillation and other potentially serious arrhythmias that would necessitate emergency cardiac interventions. Cardiac monitoring should be performed for at least the first 24 hours. (COR 1, LOE B-NR)

The effectiveness of prolonged cardiac monitoring during hospitalization after AIS to guide treatment selection for prevention of recurrent stroke is uncertain. (COR IIb, LOE C-LD)

### Class of recommendation (COR)

Class 1 (strong)

Suggested phrases for writing recommendations:

-Is recommended

-Is indicated/useful/effective/beneficial

-Should be performed/administered/other

-Comparative-Effectiveness phrases – Treatment/strategy A is recommended/indicated in preference to treatment B – Treatment A should be chosen over treatment B

Class 2b (weak)

Suggested phrases for writing recommendations:

-May/might be reasonable

-May/might be considered

-Usefulness/effectiveness is unknown/unclear/uncertain or not well established

### Level of evidence (LOE)

Level B-NR (Nonrandomized)

-Moderate-quality evidence from 1 or more well-designed, well-executed nonrandomized studies, observational studies, or registry studies

-Meta-analyses of such studies

Level C-LD (limited data)

-Randomized or nonrandomized observational or registry studies with limitations of design or execution

-Meta-analyses of such studies

-Physiological or mechanistic studies in human subjects

## Guideline: Consensus statements and recommendations from the ESO-Karolinska Stroke Update Conference, Stockholm 11–13 November 2018^4^

### Q1: What is good clinical practice in work up for suspected cardio-embolic cases?

[…] 24-h 12-lead electrocardiogram (ECG) […] (Grade A)

Continuous monitoring of heart rhythm up to 30 days is reasonable in patients with embolic stroke of undetermined aetiology despite recommended diagnostic work up to increase covert atrial fibrillation (AF) detection (Grade A). However, it remains to be firmly established that the increased detection of brief episodes of AF will lead to a reduction in stroke recurrence after adequate treatment (Grade C).

### Grade of evidence

Grade A Evidence

Strong support from randomised controlled trials and statistical reviews (at least one randomised controlled trial plus one statistical review).

Grade C Evidence

No reasonable support from randomised controlled trials, recommendations based on small randomised and/or non-randomised controlled trials evidence.

## References

1. Hindricks G, Potpara T, Dagres N, et al. 2020 ESC Guidelines for the diagnosis and management of atrial fibrillation developed in collaboration with the European Association for Cardio-Thoracic Surgery (EACTS): The Task Force for the diagnosis and management of atrial fibrillation of the European Society of Cardiology (ESC) Developed with the special contribution of the European Heart Rhythm Association (EHRA) of the ESC. *Eur Heart J* 2021; 42: 373-498. DOI: 10.1093/eurheartj/ehaa612.

2. Kleindorfer DO, Towfighi A, Chaturvedi S, et al. 2021 Guideline for the Prevention of Stroke in Patients With Stroke and Transient Ischemic Attack: A Guideline From the American Heart Association/American Stroke Association. *Stroke* 2021; 52: e364-e467. 20210524. DOI: 10.1161/STR.0000000000000375.

3. Powers WJ, Rabinstein AA, Ackerson T, et al. Guidelines for the Early Management of Patients With Acute Ischemic Stroke: 2019 Update to the 2018 Guidelines for the Early Management of Acute Ischemic Stroke: A Guideline for Healthcare Professionals From the American Heart Association/American Stroke Association. *Stroke* 2019; 50: e344-e418. 20191030. DOI: 10.1161/STR.0000000000000211.

4. Ahmed N, Audebert H, Turc G, et al. Consensus statements and recommendations from the ESO-Karolinska Stroke Update Conference, Stockholm 11-13 November 2018. *Eur Stroke J* 2019; 4: 307-317. 20190902. DOI: 10.1177/2396987319863606.
