# Supplementary material for: Large variations in atrial fibrillation screening practice after ischemic stroke and transient ischemic attack in Sweden: a survey study
Source: BMC Neurol. 2024 Apr 11;24:120. doi: 10.1186/s12883-024-03622-2 (PMC11007877; doi:10.1186/s12883-024-03622-2)
Supplement: Supplementary file 2 — Supplementary Material 2 [file 12883_2024_3622_MOESM2_ESM.docx]

# Additional file 2

# Survey on atrial fibrillation screening

To the medical supervisor at the stroke unit.

Below follows a survey to investigate clinical practice for atrial fibrillation (AF) screening at Swedish stroke units.

We are very thankful for your participation!

I hereby confirm that the answers in this survey are correct

- Yes
- No

Hospital name

________________

1. At our stroke unit the proportion of patients with ischemic stroke (without known AF) being screened for AF is:

- ≥95%
- 75-94%
- 50-74%
- <50%

Comment

________________

1. At our stroke unit the proportion of patients with TIA (without known AF) being screened for AF is:

- ≥95%
- 75-94%
- 50-74%
- <50%

Comment

________________

1. Following AF screening modalities are used:

Inpatient telemetry ECG

- Never
- Rarely
- Often
- First choice

Holter ECG

- Never
- Rarely
- Often
- First choice

Event loop recorder

- Never
- Rarely
- Often
- First choice

Handheld ECG

- Never
- Rarely
- Often
- First choice

Other, specify below

- Never
- Rarely
- Often
- First choice

Other method for AF screening

________________

Comment

________________

1. If inpatient telemetry ECG is used, which monitoring duration is most common?

- 0-24 hr
- 25-48 hr
- 49-72 hr
- >72 hr

Comment

________________

1. If inpatient telemetry ECG is used, which model is used?

________________

Comment

________________

1. If inpatient telemetry ECG is used, who does the ECG reading routinely?

- Physician responsible for the patient at the stroke unit
- Cardiologist
- Nurse at the stroke unit
- Other, specify below

Comment

________________

1. If Holter ECG is used, which monitoring duration is most common?

- 24 hr
- 48 hr
- 72 hr
- >72 hr

Comment

________________

1. If Holter ECG is used, when is the ECG recording started routinely?

- In connection to hospital stay
- The patient gets a scheduled appointment after hospital discharge
- The patient is referred

1. If event loop recorder is used, when is the ECG recording started routinely?

- In connection to hospital stay
- The patient gets a scheduled appointment after hospital discharge
- The patient is referred

1. If handheld ECG is used, when is the ECG recording started routinely?

- In connection to hospital stay
- The patient gets a scheduled appointment after hospital discharge
- The patient is referred

Comment

________________

1. Is AF screening repeated?

- Yes, routinely
- Yes, if high suspicion of cardiac embolization
- No

Specify which method/methods that are used and when:

________________

Comment

________________

1. If AF is diagnosed after hospital discharge the patient is (multiple answers possible):

(for initiation of OAC, not follow-up for frequency control etc.)

- Contacted by responsible physician at the stroke unit/stroke outpatient department
- Referred to general practitioner
- Referred to cardiologist
- Other, specify below

Other follow-up

________________

Comment

________________

1. After hospitalization for stroke/TIA, general follow-up takes place at (multiple answers possible):

- Stroke outpatient department
- Neurology outpatient department
- Internal medicine outpatient department
- General practitioner
- Other, specify below

Other unit for follow-up

________________

Comment

________________

1. When is the general follow-up scheduled? (If multiple appointments multiple answers are possible)

- <2 months
- 2-4 months
- >4 months

Comment

________________

1. Which profession is responsible for general follow-up? (multiple answers possible)

- Physician
- Nurse
- Other, specify below

Other profession

________________

1. What kind of visit for follow-up is scheduled routinely? (multiple answers possible)

- Physical visit
- Digital visit
- Telephone contact

1. Which of these is most common

- Physical visit
- Digital visit
- Telephone contact

Comment

________________
