# Supplementary material for: Large variations in atrial fibrillation screening practice after ischemic stroke and transient ischemic attack in Sweden: a survey study
Source: BMC Neurol. 2024 Apr 11;24:120. doi: 10.1186/s12883-024-03622-2 (PMC11007877; doi:10.1186/s12883-024-03622-2)
Supplement: Supplementary file 3 — Supplementary Material 3 [file 12883_2024_3622_MOESM3_ESM.docx]

# Additional file 3

Descriptive statistics for survey questions 12-17 (y-axis= number of stroke units).

12. If AF is diagnosed after hospital discharge the patient is (multiple answers possible):

(for initiation of OAC, not follow-up for frequency control etc.)

13. After hospitalization for stroke/TIA, general follow-up takes place at (multiple answers possible)

14. When is the general follow-up scheduled? (If multiple appointments multiple answers are possible)

15. Which profession is responsible for general follow-up? (multiple answers possible)

16. What kind of visit for follow-up is scheduled routinely? (multiple answers possible)

17.Which of physical visit, digital visit, telephone contact is most common?
