# Supplementary material for: Large variations in atrial fibrillation screening practice after ischemic stroke and transient ischemic attack in Sweden: a survey study
Source: BMC Neurol. 2024 Apr 11;24:120. doi: 10.1186/s12883-024-03622-2 (PMC11007877; doi:10.1186/s12883-024-03622-2)
Supplement: Supplementary file 4 — Supplementary Material 4 [file 12883_2024_3622_MOESM4_ESM.docx]

# Additional file 4

## Comparison of survey results stratified by hospital type and patient volume groups

| \| **Survey question** \| **Alternatives** \| **University and large non-university hospitals (n=31)** \| **Small non-university hospitals (n=41)** \| **p-value** \| \| --- \| --- \| --- \| --- \| --- \| \| Proportion of screened patients ischemic stroke \| <75% vs. ≥75% \| 2 (6%) vs. 29 (94%) \| 1 (2%) vs. 40 (98%) \| 0.574* \| \| Proportion of screened patients TIA \| <75% vs. ≥75% \| 3 (10%) vs. 28 (90%) \| 2 (5%) vs. 39 (95%) \| 0.646* \| \| Use of inpatient telemetry ECG \| Never/rarely vs. often/first choice \| 7 (23%) vs. 24 (77%) \| 6 (15%) vs. 35 (85%) \| 0.385 \| \| Use of holter ECG \| Never/rarely vs. often/first choice \| 16 (52%) vs. 15 (48%) \| 26 (63%) vs. 15 (37%) \| 0.315 \| \| Use of ELR \| Never/rarely vs. often/first choice \| 30 (97%) vs. 1 (3%) \| 41 (100%) vs. 0 (0%) \| 0.431* \| \| Use of handheld ECG \| Never/rarely vs. often/first choice \| 16 (52%) vs. 15 (48%) \| 28 (68%) vs. 13 (32%) \| 0.151 \| \| Use of other AF screening method \| Never/rarely vs. often/first choice \| 15 (100%) vs. 0 (0%) missing n=16 \| 24 (96%) vs. 1 (4%) missing n=16 \| 1.000* \| \| Monitoring duration for inpatient telemetry ECG \| ≤48hr vs. >48hr \| 22 (85%) vs. 4 (15%) \| 34 (83%) vs. 7 (17%) \| 1.000* \| \| Monitoring duration for holter ECG \| 24/48hr vs. 72/>72hr \| 24 (80%) vs. 6 (20%) \| 27 (77%) vs. 8 (23%) \| 0.780 \| \| Start of ECG recording for holter \| Hospital stay/scheduled vs. referred \| 9 (30%) vs. 21 (70%) \| 15 (43%) vs. 20 (57%) \| 0.284 \| \| Start of ECG recording for ELR \| Hospital stay/scheduled vs. referred \| 1 (8%) vs. 12 (92%) \| 4 (15%) vs. 23 (85%) \| 1.000* \| \| Start of ECG recording for handheld ECG \| Hospital stay/scheduled vs. referred \| 15 (65%) vs. 8 (35%) \| 17 (63%) vs. 10 (37%) \| 0.869 \| \| Repeated AF screening \| Yes/No \| 26 (84%) vs. 5 (16%) \| 36 (88%) vs. 5 (12%) \| 0.736* \| \| **Survey question** \| **Alternatives** \| **Low patient volume (n=36)** \| **High patient volume (n=36)** \| **p-value** \| \| Proportion of screened patients ischemic stroke \| <75% vs. ≥75% \| 1 (3%) vs. 35 (97%) \| 2 (6%) vs. 34 (94%) \| 1.000* \| \| Proportion of screened patients TIA \| <75% vs. ≥75% \| 2 (6%) vs. 34 (94%) \| 3 (8%) vs. 33 (92%) \| 1.000* \| \| Use of inpatient telemetry ECG \| Never/rarely vs. often/first choice \| 7 (19%) vs. 29 (81%) \| 6 (17%) vs. 30 (83%) \| 0.759 \| \| Use of holter ECG \| Never/rarely vs. often/first choice \| 20 (56%) vs. 16 (44%) \| 22 (61%) vs. 14 (39%) \| 0.633 \| \| Use of ELR \| Never/rarely vs. often/first choice \| 36 (100%) vs. 0 (0%) \| 35 (97%) vs. 1 (3%) \| 0.500* \| \| Use of handheld ECG \| Never/rarely vs. often/first choice \| 27 (75%) vs. 9 (25%) \| 17 (47%) vs. 19 (53%) \| **0.016** \| \| Use of other AF screening method \| Never/rarely vs. often/first choice \| 22 (96%) vs. 1 (4%)  missing n= 13 \| 17 (100%) vs. 0 (0%)  missing n=19 \| 0.575* \| \| Monitoring duration for inpatient telemetry ECG \| ≤48hr vs. >48hr \| 29 (83%) vs. 6 (17%) \| 27 (84%) vs. 5 (16%) \| 0.867 \| \| Monitoring duration for holter ECG \| 24/48hr vs. 72/>72hr \| 26 (84%) vs. 5 (16%) \| 25 (74%) vs. 9 (27%) \| 0.311 \| \| Start of ECG recording for holter \| Hospital stay/scheduled vs. referred \| 14 (45%) vs. 17 (55%) \| 10 (29%) vs. 24 (71%) \| 0.189 \| \| Start of ECG recording for ELR \| Hospital stay/scheduled vs. referred \| 3 (13%) vs. 21 (88%) \| 2 (13%) vs. 14 (88%) \| 1.000* \| \| Start of ECG recording for handheld ECG \| Hospital stay/scheduled vs. referred \| 11 (52%) vs. 10 (48%) \| 21 (72%) vs. 8 (28%) \| 0.145 \| \| Repeated AF screening \| Yes/No \| 33 (92%) vs. 3 (8%) \| 29 (81%) vs. 7 (19%) \| 0.307* \| |
| --- | --- | --- | --- | --- | --- | --- | --- | --- | --- | --- | --- | --- | --- | --- | --- | --- | --- | --- | --- | --- | --- | --- | --- | --- | --- | --- | --- | --- | --- | --- | --- | --- | --- | --- | --- | --- | --- | --- | --- | --- | --- | --- | --- | --- | --- | --- | --- | --- | --- | --- | --- | --- | --- | --- | --- | --- | --- | --- | --- | --- | --- | --- | --- | --- | --- | --- | --- | --- | --- | --- | --- | --- | --- | --- | --- | --- | --- | --- | --- | --- | --- | --- | --- | --- | --- | --- | --- | --- | --- | --- | --- | --- | --- | --- | --- | --- | --- | --- | --- | --- | --- | --- | --- | --- | --- | --- | --- | --- | --- | --- | --- | --- | --- | --- | --- | --- | --- | --- | --- | --- | --- | --- | --- | --- | --- | --- | --- | --- | --- | --- | --- | --- | --- | --- | --- | --- | --- | --- | --- | --- |

Table S1. The table shows p-values (far right) from chi-square/fisher exact analysis of data by hospital type and patient volume groups.

**Bold**=significant, *fisher exact
